# Successful treatment of refractory pyoderma gangrenosum with risankizumab in a 57-year-old patient: A case report

**DOI:** 10.1177/2050313X251352130

**Published:** 2025-06-20

**Authors:** Damy Horth, Nora Assouyat, Isabelle Auger

**Affiliations:** 1 Department of Dermatology, Université Laval, Québec, QC, Canada; 2 Department of Medicine, Université Laval, Québec, QC, Canada

**Keywords:** dermatology, pyoderma gangrenosum, refractory pyoderma gangrenosum, risankizumab, neutrophilic dermatoses

## Abstract

Pyoderma gangrenosum is a neutrophilic dermatosis characterized by rapidly progressing inflammatory skin lesions. It is often associated with underlying systemic conditions, such as inflammatory bowel disease and rheumatoid arthritis. Patients typically present with erythematous papules and pustules that rapidly evolve into painful ulcers, most commonly affecting the lower extremities. In this case report, we describe a 57-year-old female patient with multirefractory pyoderma gangrenosum localized to the lower left leg. The diagnosis was confirmed based on clinical and histopathological features, with a skin biopsy showing compatible findings. Initial treatments, including topical therapies (high-potency steroids, dapsone, and calcineurin inhibitors) and conventional systemic immunosuppressive therapies (corticosteroids and tumor necrosis factor inhibitors), failed to produce significant improvement. However, treatment with risankizumab, an interleukin-23 inhibitor, resulted in substantial ulcer healing over a few weeks and ultimately led to complete resolution.

## Introduction

Pyoderma gangrenosum (PG) is an inflammatory skin condition characterized by painful, rapidly progressing ulcerative lesions that evolve from erythematous papules and pustules.^1^ While the exact pathogenesis is not fully understood, it is believed to involve a genetic predisposition and dysregulation of the immune response, with neutrophil-mediated tissue damage and inflammation playing a central role.^2^ Interleukin-23 (IL-23) appears to be particularly involved, inducing, and maintaining a T helper 1 (Th1) and Th17 cell response, which, in turn, drives the secretion of pro-inflammatory cytokines (including tumor necrosis factor-α (TNF-α), IL-1, IL-8, IL-17, and IL-23), thereby contributing to neutrophil recruitment.^3^ PG can be associated with underlying systemic diseases, but many cases are idiopathic. The most common associated conditions include inflammatory bowel disease (IBD; such as Crohn’s disease and ulcerative colitis), arthritis, and hematologic malignancies.^2^

The diagnosis of PG is based on clinical and histopathological features. It is crucial to exclude other conditions, as misdiagnosis is not uncommon and may expose patients to unnecessary treatment risks.^4^

Initial management typically involves local therapy for milder presentations. The most common treatments include high-potency topical steroids (e.g., clobetasol) or calcineurin inhibitors (e.g., tacrolimus). Intralesional corticosteroids, oral antibiotics, colchicine, and dapsone can be added as adjunctive therapies. If there is no improvement, or if the initial presentation involves larger or multifocal lesions, systemic corticosteroids and/or cyclosporine are commonly used due to their rapid effects in halting ulcer progression. Biologic agents, such as infliximab or adalimumab (TNF-α inhibitors), are often introduced to sustain immune suppression. However, managing PG can be challenging, particularly in refractory cases.^5^ The overexpression of IL-23 implies that risankizumab, a monoclonal antibody targeting the IL-23 p19 subunit, may be an option for treatment, as demonstrated in this case report.^6^

## Case

**Figure 1.**
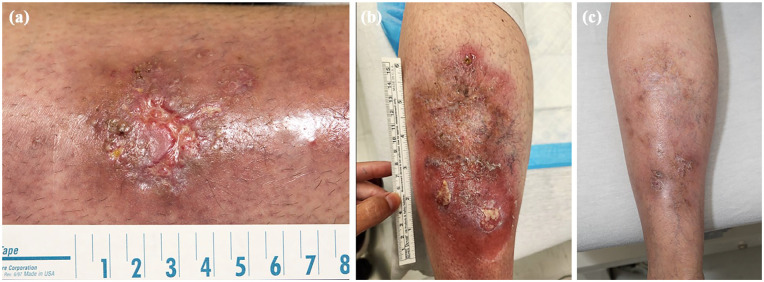
(a) At baseline. (b) Relapse of pyoderma gangrenosum despite treatment with adalimumab (TNF inhibitor), topical, and systemic therapies. (c) Complete resolution of PG after treatment with risankizumab. PG: pyoderma gangrenosum; TNF: tumor necrosis factor.

A 57-year-old female patient presented to the dermatology department in 2017 with an ulcerative lesion on her left lower leg that was refractory to systemic antibiotics, suggestive of PG (Figure 1(a)). No other areas of the body were involved. Her medical history included stage 2 pulmonary sarcoidosis, type 2 diabetes, hypertension, dyslipidemia, hidradenitis suppurativa (HS), and polycythemia secondary to smoking. An incisional skin biopsy revealed deep dermal neutrophilic infiltrates with abscess formation. Bacterial, mycobacterial, and fungal cultures were negative. No associations with IBD, arthritis, or malignancy were identified, despite an extensive work-up that has been monitored over the years. A diagnosis of pyoderma gangrenosum, acne, and suppurative hidradenitis syndrome secondary to a PSTPIP1 gene mutation was declined, as the patient had never experienced acne lesions, and the HS was mild.

From December 2017 to January 2023, multiple cycles of oral prednisone were administered at a dosage of 1 mg/kg/day, followed by gradual tapering. Concurrently, topical therapies including tacrolimus ointment, clobetasol ointment, and localized wound care were used. Despite the administration of minocycline, doxycycline, and oral dapsone, optimal control of PG was not achieved, with recurrent episodes and persistent inflammation over the years. Cyclosporine was contraindicated due to the patient’s comorbidities.

In January 2023, subcutaneous adalimumab was initiated at a dosage of 40 mg weekly. An initial clinical response was observed, but the patient subsequently developed fatigue and severe palmoplantar pustulosis. In June 2023, a new painful erythematous nodule appeared on the anterior surface of the right shin, representing a flare of PG despite ongoing treatment with adalimumab. Adalimumab was discontinued, and oral prednisone, along with the previously used topical and systemic medications, including dapsone, was reintroduced. Colchicine was also trialed but discontinued after a few doses due to poor tolerance. Unfortunately, relapses occurred (Figure 1(b)).

In January 2024, risankizumab was initiated at an initial dose of 150 mg subcutaneously at week 0, followed by a second dose of 150 mg after 4 weeks, and then 150 mg every 12 weeks, in combination with oral dapsone (150 mg/day) and regular wound care. By the end of March 2024, after two doses of risankizumab, the ulceration had nearly resolved. At the follow-up in early September 2024, PG had completely healed (Figure 1(c)). There was no pain, and dapsone was reduced to 100 mg/day, with a further planned reduction to 50 mg/day. The patient was still doing well at the time of submission of this paper.

## Discussion

PG is a rare, inflammatory skin disorder classified as one of the neutrophilic dermatoses. The classic variant is characterized by ulcerative lesions with undermined, violaceous borders that progress rapidly. A high level of clinical suspicion is required, and exclusion of other mimicking conditions is crucial to avoid misdiagnosis and inappropriate treatment. Histopathological analysis through skin biopsy, in conjunction with clinical presentation, is essential to confirm the diagnosis, although the biopsy findings can be nonspecific.^7^

While the exact pathogenesis of PG is not fully understood, immune dysregulation involving Th1/Th17 pathways and overexpression of several cytokines, such as IL-23, appears to play a key role in neutrophil-mediated inflammation and tissue damage, thereby establishing promising new therapeutic targets.^8^

Conventional therapies include topical high-potency corticosteroids, topical calcineurin inhibitors, systemic antibiotics, colchicine, and dapsone for milder cases. For more severe cases, systemic corticosteroids and other immunosuppressive drugs, such as cyclosporine, are usually administered. Biologic agents, particularly TNF-α inhibitors like infliximab and adalimumab, have shown efficacy in controlling recalcitrant cases of PG.^9^ However, some cases remain unresponsive to standard therapy, highlighting the need to target other players in the inflammatory cascade, as demonstrated by our case, where the use of an anti-IL-23 agent led to the rapid healing of a refractory PG lesion.

In conclusion, this case underscores the importance of individualized treatment strategies and suggests that risankizumab may be an effective option for patients with refractory PG who have failed multiple lines of therapy, including TNF-α inhibitors. Ongoing follow-up is recommended to monitor for potential relapse and adjust therapy accordingly.
